# Machine learning-based optimization of pre-symptomatic COVID-19 detection through smartwatch

**DOI:** 10.1038/s41598-022-11329-y

**Published:** 2022-05-12

**Authors:** Hyeong Rae Cho, Jin Hyun Kim, Hye Rin Yoon, Yong Seop Han, Tae Seen Kang, Hyunju Choi, Seunghwan Lee

**Affiliations:** 1 Department of Intelligence and Communication Engineering, Geyongsang National University, Jinju, 52828 South Korea; 2 Department of Ophthalmology, Institute of Health Sciences, Gyeongsang National University College of Medicine and Gyeongsang National University Changwon Hospital, Changwon, 51472 South Korea; 3 Deepnoid Inc., Seoul, South Korea

**Keywords:** Biological techniques, Software, Infectious diseases, Influenza virus, Viral infection

## Abstract

Patients with weak or no symptoms accelerate the spread of COVID-19 through various mutations and require more aggressive and active means of validating the COVID-19 infection. More than 30% of patients are reported as asymptomatic infection after the delta mutation spread in Korea. It means that there is a need for a means to more actively and accurately validate the infection of the epidemic via pre-symptomatic detection, besides confirming the infection via the symptoms. Mishara et al. (Nat Biomed Eng 4, 1208–1220, 2020) reported that physiological data collected from smartwatches could be an indicator to suspect COVID-19 infection. It shows that it is possible to identify an abnormal state suspected of COVID-19 by applying an anomaly detection method for the smartwatch’s physiological data and identifying the subject’s abnormal state to be observed. This paper proposes to apply the One Class-Support Vector Machine (OC-SVM) for pre-symptomatic COVID-19 detection. We show that OC-SVM can provide better performance than the Mahalanobis distance-based method used by Mishara et al. (Nat Biomed Eng 4, 1208–1220, 2020) in three aspects: earlier (23.5–40% earlier) and more detection (13.2–19.1% relative better) and fewer false positives. As a result, we could conclude that OC-SVM using Resting Heart Rate (RHR) with 350 and 300 moving average size is the most recommended technique for COVID-19 pre-symptomatic detection based on physiological data from the smartwatch.

## Introduction

More than 5 million people have died by the end of 2021 since the severe acute respiratory syndrome COVID-19 virus outbreaks in 2020 as a global pandemic (https://covid19.who.int). The disease caused by this virus typically presents as a lower respiratory infection, followed by many atypical presentations^2^. In particular, a previously unexposed population has been affected by the high transmissibility of this virus and still undergone a significant health challenge even after recovering from the diseases caused by this virus.

Recently, COVID-19 infection by asymptomatic and pre-symptomatic patient keeps increasing. In particular, OMICRON, a mutant of COVID-19, accelerates the spread of COVID-19. In the case of South Korea, one out of five cases of COVID-19 infection is the patient infected by asymptomatic patients. The disease is highly contagious. According to one model, it begins to be contagious 2.3 days and peaks 0.7 days prior to the onset of symptoms. Thus, a great deal of effort is underway to potentially diagnose COVID-19 early^2^.

Some studies have reported that consumer devices, such as smartwatches, can effectively detect signs of illness by measuring the patient’s respiration rate, heart rate, and heart rate variability (HRV). A 1 $^\circ$C rise in body temperature can increase heart rate by 8.5 beats per minute (b.p.m.) on average^3^, and breathing rate increases when the patient has a fever^4^. HRV, the variability of the time between successive heartbeats, can be used as a valuable indicator of the non-invasive probe of the autonomic nervous system^5,6^. The lowered values of HRV are indicative of increased mortality^7^ and may provide early diagnosis of infection^7^. In particular, measuring the resting heart rate or heart rate during sleep can therefore be a practical diagnostic means. In addition to those above studies, Mishara et al.^1^ and Aravind et al.^2^ show that the variability of heart rate and steps measured by smartwatch can predict the onset of COVID-19. In particular, they used human steps and heart rate as a health metric to predict the onset of COVID-19. Interestingly, it showed effectiveness by detecting 63% of COVID-19 cases before symptom onset. The technique that Mishara et al.^1^ used is the anomaly detection based on Mahalanobis distance, one of the classical techniques for finding multivariate outliers, which indicates unusual combinations of two or more variables.

This paper reports optimization of COVID-19 detection of the anomaly detection using health metrics from the smartwatch by using a machine learning, One Class-Support Vector Machine (OC-SVM). OC-SVM is an unsupervised algorithm that learns a decision function for novelty detection: classifying new data as similar or different to the training set. This paper shows the improvement of the anomaly detection performance of pre-symptomatic COVID-19 detecting in two aspects by using OC-SVM. We also apply MD-MCD and OC-SVM for COVID-19 negative participants to show the suppression of false positives.

## Results

This section provides three results from applying our approach for the same data of Mishara et al.^1^: (1) Early detection of COVID-19, (2) shortening the minimum period of sampling data for valid anomaly detection, and (3) suppressing false-positive anomaly detection. In this study, our goal of applying OC-SVM is to detect COVID-19 sooner and find more valid anomaly detection instances by proposing an earlier time point based on a smaller size of datasets.

Table 1 presents abbreviations of this paper and their explanations. The abbreviations combine sampling models (e.g. RHR or HROS) and methods (MD or OC-SVM).

**Table 1:** Abbreviations.

Abbreviation	Explanations
RHR-MD	Resting heart rate mahalanobis distance
RHR-OC-SVM	Resting heart rate one class support vector machine
HROS-MD	Heart rate of steps mahalanobis distance
HROS-OC-SVM	Heart rate of steps one class support vector machine

### Optimization of anomaly detection of COVID-19

In this sub-section, we compare the number of detected outliers and the time point of reporting anomaly obtained by applying OC-SVM to the RHR and HROS models of Mishara et al.^1^.

**Table 2:** A summary of Table 3.

Comparison aspects	Models/methods			
	RHR		HROS	
	MD-MCD	OC-SVM	MD-MCD	OC-SVM
The number of smartphone-detected COVD-19 positives	15	21	17	24
The average of outliers per COVD-19 positive (for 3 weeks)	21.66	20.76	25.31	21.41
The average of time distances between the first outlier occurrence and participant’s reporting of COVID-19 symptoms onset (early detection time)	7 day 2 h (170 h)	11 day 2 h (266 h)	11 day (168 h)	11 day 12 h (276 h)

In the rest of this paper, we compare four combinations of model-method (RHR-MD, RHR-OC-SVM, HROS-MD, and HROS-OC-SVM). RHR-MD denotes a case in which MD-MCD method is applied to RHR model, and RHR-OC-SVM indicates a case in which OC-SVM is applied to RHR model.

**Table 3:**
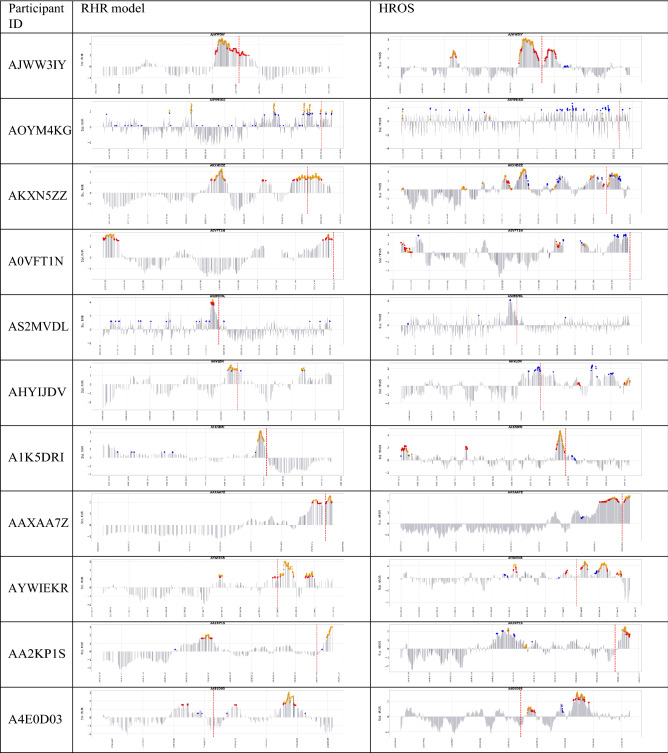

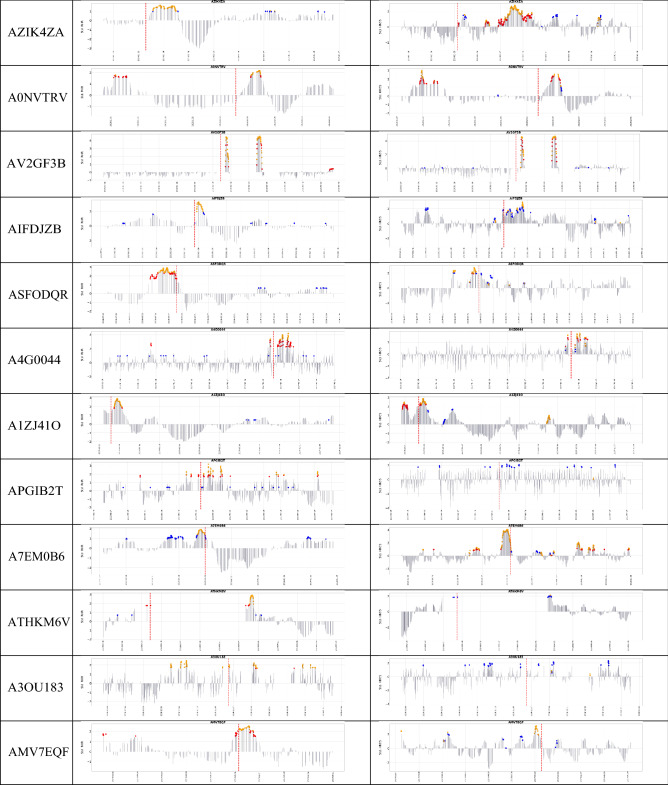

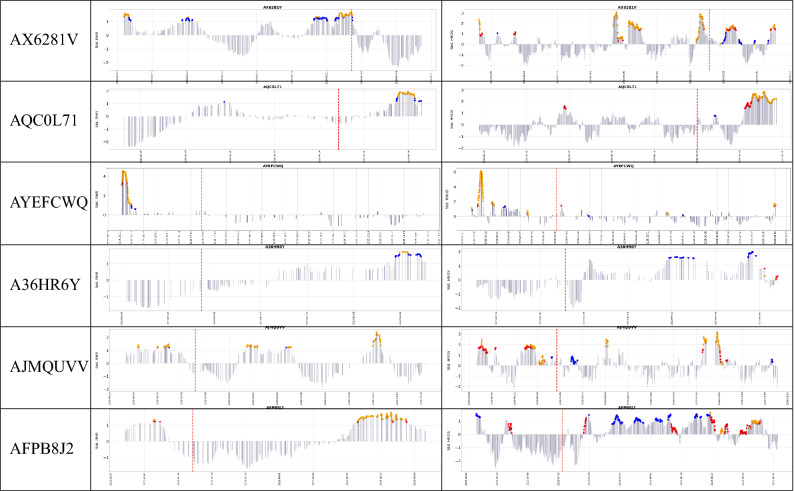
Comparison of anomaly detection instances of RHR-MD, RHR-OC-SVM, HROS-MD, and HROS-OC-SVM: blue dots are outliers found by OC-SVM, red dots are outliers found by MD-MCD, and orange dots are outliers found by both OC-SVM and MD-MCD.

Table 3 shows the results of applying the combinations of MD-MCD and OC-SVM and RHR and HROS models for 29 participants of COVID-19 positive. It marks anomaly detection points, i.e., outliers, three weeks before participants report COVID-19 symptoms onset and identifies four combinations of MD-MCD and OC-SVM, RHR, and HROS models with three colored dots. Blue dots are outliers found by OC-SVM, red dots are outliers found by MD-MCD, and orange dots are outliers found by OC-SVM and MD-MCD. Table 2 summarizes Table 3 in three aspects: the number of participants from which methods found outliers, the average number of outliers per participant, and the average time distance from the first time point of outlier occurrence to the time point for participants to report COVID-19 symptoms onset.

First, the results of applying the RHR model to MD-MCD and OC-SVM methods are as follows: MD-MCD using RHR model (RHR-MD) in Mishara et al.^1^ detects outliers from 15 participants out of 32 COVID-19 positive participants, excluding three error cases, and the average of the detected outliers per participant was 21.66%. OC-SVM using the RHR model (RHR-OC-SVM) detects outliers from 21 participants and finds outliers from 6 more participants (AYWIEKR, A4E0D03, ATHKM6V, AQC0L71, AJMQUVV, AFPB8J2) than Mishara et al.^1^. The number of outliers found by RHR-OC-SVM averages 20.76 per patient for the same participants. It is noteworthy that OC-SVM detects outliers from more patients than MD-MCD, but the average number of outliers found for 29 patients is about 0.9 less than MD-MCD. The time differences between the participant’s symptom (occurrence) reporting date and the first outlier occurrence are 7 days 2 h for MD-MCD and 11 days 2 h for OC-SVM, respectively. OC-SVM using RHR model reports the outlier about 4 days earlier on average than MD-MCD.

Second, the results of applying HROS model to MD-MCD and OC-SVM methods are as follows: Mishara et al.^1^ finds outliers from 17 participants out of 29 participants, and the average number of outliers per participant is 25.31. OC-SVM using HROS model (HROS-OC-SVM) finds outliers from 24 participants, of which 7 are participants (AS2MVDL, AHYIJDV, A0NVTRV, AV2GF3B, APGIB2T, ATHKM6V, A3OU183) from which Mishara et al.^1^ finds no outliers. The number of outliers found by HROS-OC-SVM averages 21.41 per participant. It is noteworthy that OC-SVM detects more outliers than MD-MCD and generates 3.9 fewer outliers on average, the same as RHR-OC-SVM. The time point differences of the first outlier occurrence from the time points for participants to report COVID-19 symptom onset are 11 days for MD-MCD and 11 days 12 h for OC-SVM on average, respectively. From these two comparisons, we could conclude that OC-SVM using both RHR or HROS models detects anomalies from more participants earlier than MD-MCD.

**Figure 1:**
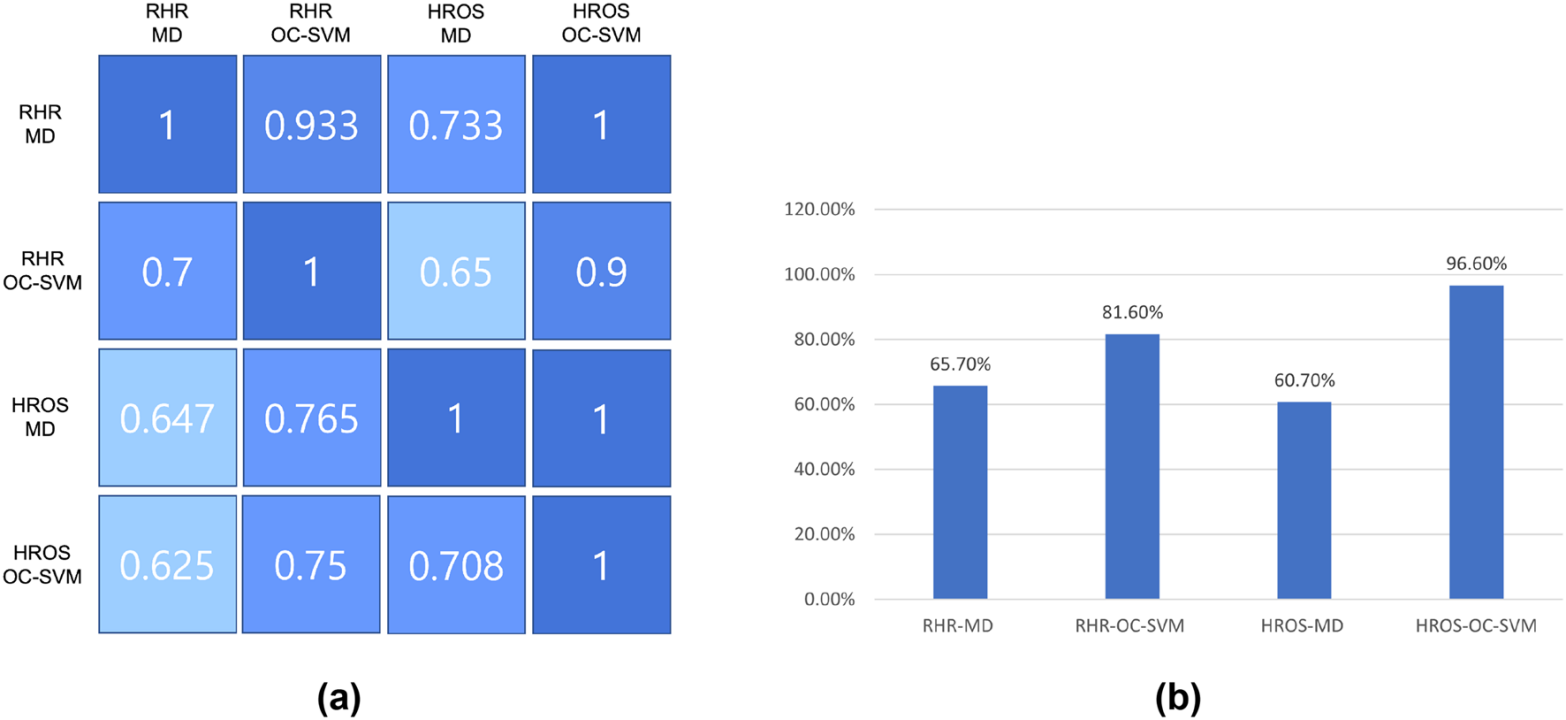
(**a**) A confusion matrix of the relative accuracy rates of the anomaly detection techniques. (**b**) The average the relative accuracy rate of each technique w.r.t the other techniques.

Figure 1a is a confusion matrix of the relative detection accuracy that shows a relative detection ability of four techniques with respect to (w.r.t) each other. The value of a cell in Fig. 1a is the ratio of detection cases estimated by the combinations of models and methods on the *x*-axis to the detection cases confirmed by the combination of models and methods on the *y*-axis. For example, 0.933 in the cell of the first row and the second column indicates that the ratio of the cases that RHR-MD estimates COVID-19 positive is 0.933 for all the cases that RHR-OC-SVM confirms COVID-19 positive. Actually, for the 15 cases confirmed by RHR-MD, RHR-OC-SVM finds anomalies from 14 cases. RHR-MD detects 70%, 64.7%, and 62.5% of all COVID-19 confirmed cases found by RHR-OC-SVM, HROS-MD, and HROS-OC-SVM; thus the average ratio of the relative detection accuracy of RHR-MD is 66.7%. RHR-OC-SVM detected 93.3%, 76.5%, and 75% of all confirmed cases found in RHR-MD, HROS-MD, and HROS-OC-SVM, respectively. It has a relative detection accuracy of 81.6% compared to all other techniques found.

Figure 1b shows the average relative detection accuracy rate of each technique w.r.t the other techniques: RHR-OC-SVM and HROS-OC-SVM using RHR-MD and HROS-MD models show the relative detection accuracy 15–20% and 30–35%, respectively, higher than MD-MCD methods using the same models. Note that it does not take into account false positives. We will explain the performance evaluation of the relative detection accuracy rate, including consideration of false positives in section “Optimization of false-positive suppression.”

### Optimization of the initial detection period

The minimum anomaly detection period (MADP) refers to a period when the minimum data required for initial effective anomaly detection is collected. The shorter the MADP period, the faster the detection begins to be meaningful. Our experiments minimize MADP by minimizing the data window size required when calculating the moving average of anomaly detection for faster detection of COVID-19. The data window size is called moving average size in this paper. A moving average is a set of averages computed over a specific size *k* of a subset of time series data by moving a specific time step. The reason for using a moving average is that it can easily distinguish between normal time series data and anomalous data by showing the trend of data with many abnormalities, such as heavy, irregular fluctuations. Reducing the moving average size should not affect the accuracy of detection i.e., detection rate and false-positive rate. An optimal moving average size *k* maximizes the anomaly detection with the smallest *k* without increasing the false positives.

This experiment compares the number of outliers reported three weeks prior to participants’ reporting of COVID-19 symptom onset and the time point of the first outlier occurrence, depending on the moving average size. In this paper, we find an optimal *k* to group a dataset by reducing the value by 50 from $k=400$ used in Mishara et al.^1^. As mentioned earlier, shrinking the value of *k* should not decrease the rate of anomaly detection or increase the rate of false positives. We combine 12 techniques (12 combinations of models, methods, and moving average sizes) by applying three moving average sizes (400, 350, and 300) to each technique of RHR-MD, RHR-OC-SVM, HROS-MD, and HROS-OC-SVM. RHR-MD-400 and HROS-MD-400 are the techniques used by Mishara et al.^1^. The number following the abbreviations in Table 1 denotes a window size of sampling data. For example, RHR-MD-400 represents the technique that combines the Resting Heart Rate (RHR) model, Mahalanobis Distance method using the moving average size of 400.

**Figure 2:**
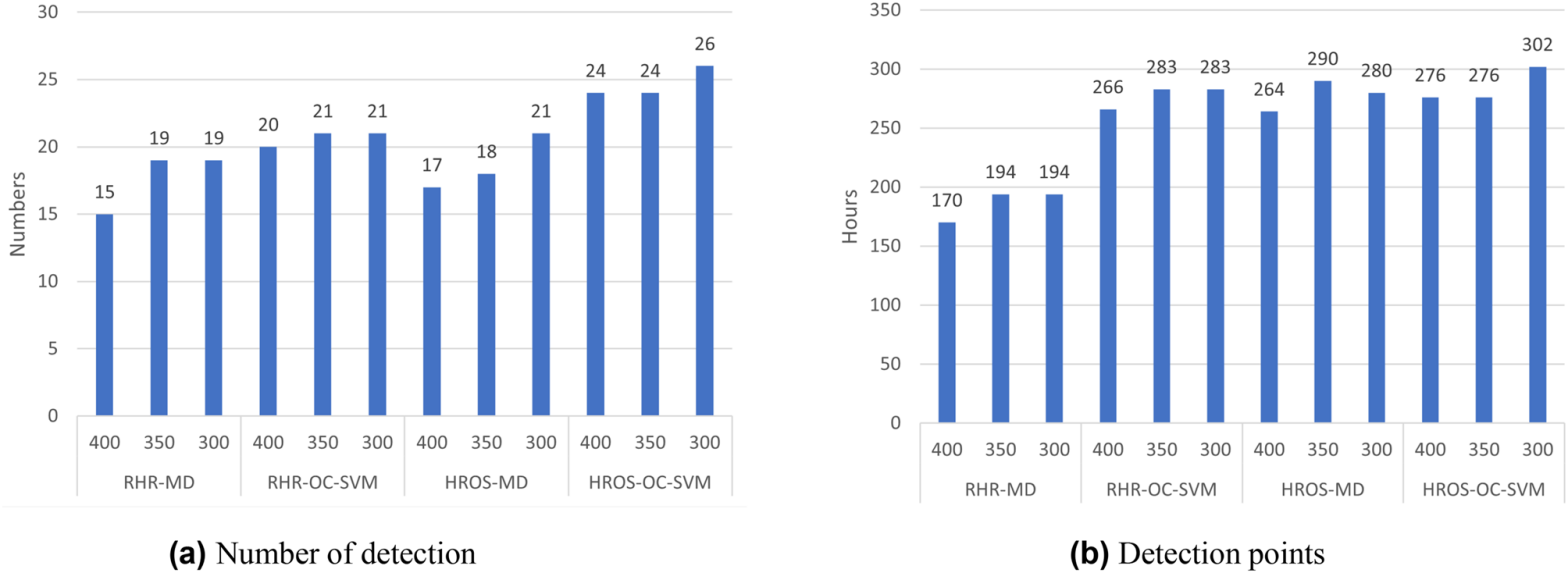
Variations of anomaly detection performances by moving averages.

For three different moving average size *k*, we apply each combination of models and methods for the same data sets. Figure 2 shows (1) the number of COVID-19 confirmed cases detected by each model-method the 3 weeks prior to participant’s reporting of COVID-19 symptoms onset, and (2) the average distances between the first anomaly detection points and the time points of reporting COVID-19 symptoms by each participant.

A technique i.e., a combination of model-technique-moving average *k* can be said to be better than others when it finds more confirmed cases from the same data as soon as possible. In the previous section, for $k=400$, RHR-OC-SVM and HROS-OS-SVM reported more COVID-19 confirmations earlier than the other two model techniques. For $k=350$, it can be seen that the overall number of infected people increases, and anomalies are detected earlier. In particular, in the case of MD-MCD, performance improvement can be seen in both models, and OC-SVM can also see a slight performance improvement in RHR model. In the case of a moving average of $k = 300$, only the HROS-OC-SVM with $k=350$ can see an improvement in the number of infected people or the performance at the time of detection. If the size of the moving average *k* is reduced in this way, the moving average (data) size may be biased toward specific data. Therefore, in this paper, the false positive rate of non-infected persons is corrected. The *k* value with the smallest moving average size and the false positive rate is selected.

**Figure 3:**
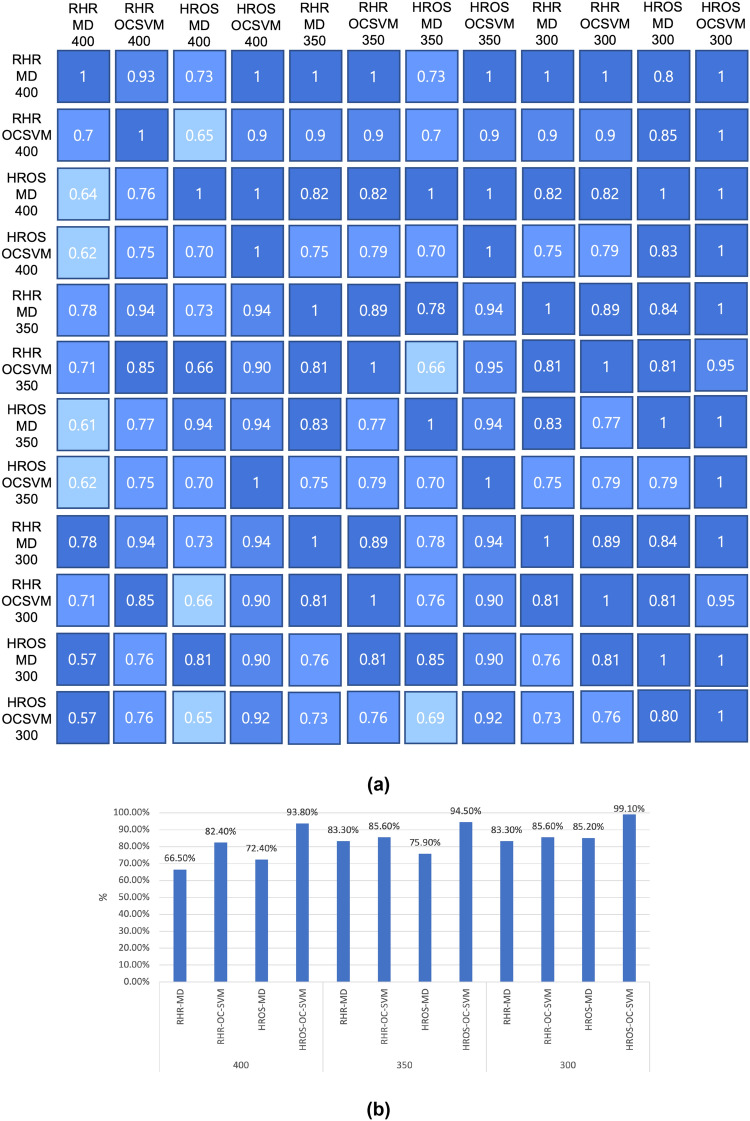
(**a**) A confusion matrix of the relative accuracy rate of anomaly detection for each combination of models, methods, and the moving average sizes. (**b**) The average of the relative accuracy rate of each technique w.r.t. the other techniques.

Figure 3a is a relative detection accuracy confusion matrix comparing the detection accuracy of the number of patients with respect to each other for various combinations of model-technique-moving average *k*-values. The value of each cell is the ratio of the number of patients detected by the model-method-k value on the x-axis to the number of patients detected by the model-method-k value on the y-axis.

Figure 3b shows the average of the performance of comparison (detection) accuracy for anomalies detected by other methods except for the corresponding method for each model-method-k value combination based on summarizes Fig. 3a. For example, to obtain the performance of RHR-MD with k = 400, it is the average of the sum of the accuracy of 11 methods except for the RHR-MD-400 method in the first row and first column of Fig. 3b. Therefore, the mean change in accuracy compared to confirmed patients increased by an average of 6.05% at k = 350 compared to k = 400, and by an average of 9.53% at k = 300 compared to k = 400. In Fig. 3b, it can be seen that the overall performance improves as the value of k decreases. However, it can be seen that HROS-OC-SVM rises from 93.8 to 99.1%, and RHR-OC-SVM has a comparison accuracy of 85.6% at k = 350, and then the comparison accuracy does not increase further at k = 350. Other MD-MCD methods also show that the comparison accuracy is improved from at least about 12% to about 18%.

**Figure 4:**
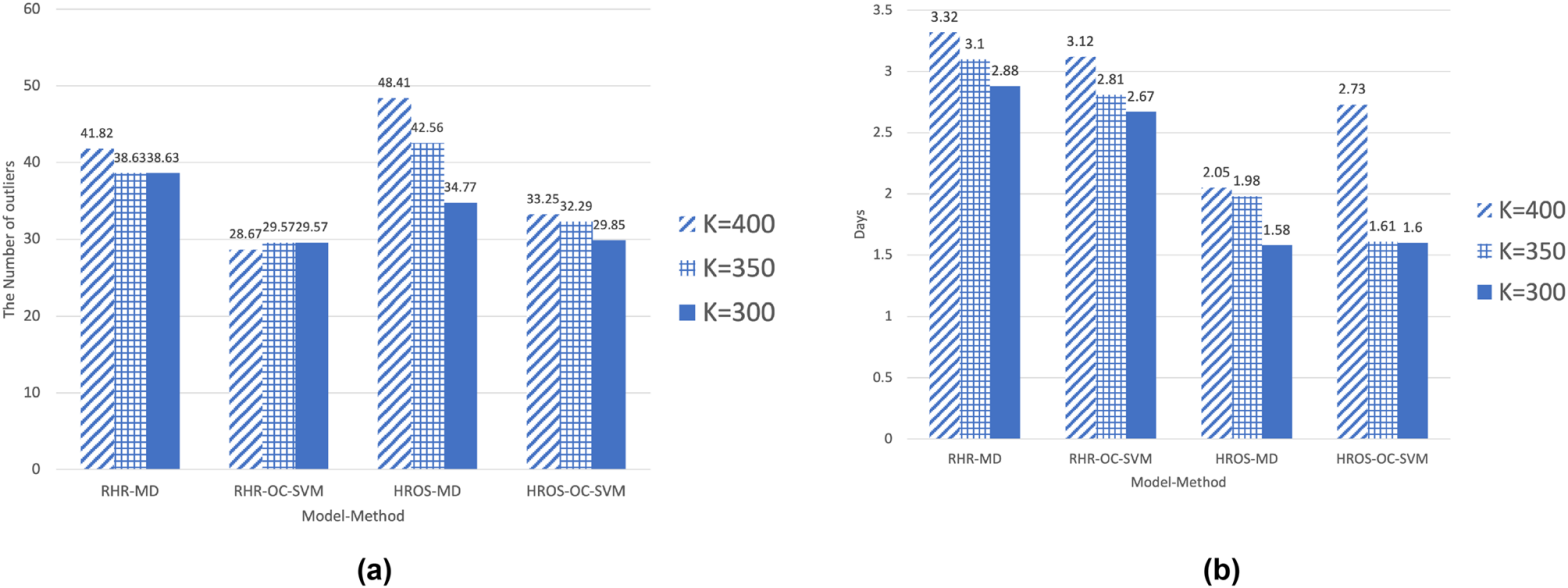
(**a**) The total number of outliers produced by each techniques (**b**) The minimum training periods of each technique.

Figure 4a shows the average number of outliers over the three weeks after symptom onset for 29 confirmed cases of COVID-19. The *x*-axis is the anomaly detection method, and the *y*-axis is the average number of outliers produced by each technique. Diagonal striped bars for k = 400, grid bars for k = 350, blue bars for k = 300.

From Fig. 4a, RHR-OC-SVM and HROS-OC-SVM detect fewer outliers but detect outliers from more participants than RHR-MD-MCD and HROS-MD. In particular, we observe that RHR-OC-SVM is not significantly affected by the small moving average size.

The moving average size affects the MADP. That is, if the size of the moving average is large, the MADP becomes longer, which may delay the initial detection of anomalies. Thus, RHR-OC-SVM is relatively less affected by the moving average size and more stable than other methods. Compared to the other methods, it less suffers the overfitting issue where a model is biased to specific data. Also, RHR-OC-SVM finds more COVID-19 infected ones but reported fewer outliers than MD-MCD using the same data sizes and periods. It might imply that RHR-OC-SVM has fewer false positives compared to other techniques.

Figure 4b shows the minimum learning time required for the techniques of 12 combinations of model-technique-moving average *k* to report outliers based on the data of 29 confirmed COVID-19 patients. The minimum learning time can be computed by repeatedly setting the data size and testing until a technique under testing produces no error in detecting outliers. In this graph, techniques using HROS generally require more minimum learning time. The reason is that RHR treats only the data in this case where the steps are 0 as valid data, and HROS treats the collected data as valid data regardless of the steps. Therefore, it takes more time to collect enough data of the RHR model for the anomaly detection, so the minimum learning time for the technique using the RHR model is longer than the technique using the HROS model. Nevertheless, once the method’s learning via the RHR model is over, the outliers emerge faster and more accurately than the HROS model.

In the next section, we evaluate the performance of each method against false positives through the results of 74 non-infected patients with COVID-19 for each technique of model-method- moving average *k*.

### Optimization of false-positive suppression

In this section, we present the results of applying 12 techniques combining the model-method-moving average *k* to 74 COVID-19 negative participants to see the ability for each technique to suppress the false-positive detection.

From Fig. 3b, HROS-OC-SVM technique using the moving average size $k=300$ shows the best anomaly detection rate, 99.1%. However, it may not be suitable for distinguishing uncertainties because it becomes too sensitive to the trend of the data variation due to a small moving average size of data, and the false positive detection rate also becomes high. Therefore, in this section, we aim to find the model-method-moving average *k* with the least outliers for the data of 74 COVID-19 negative participants.

**Figure 5:**
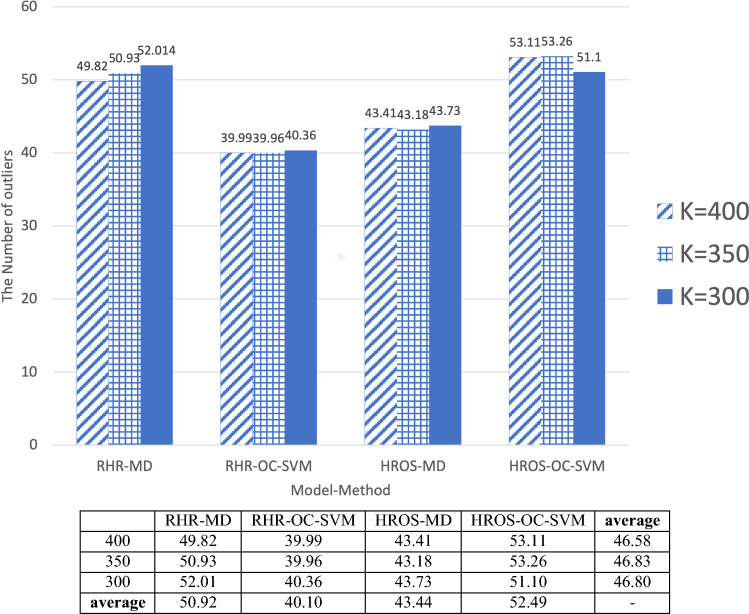
The total number of outliers from COVID-19 negative participants.

Figure 5 plots the number of outliers detected by applying 12 methods combining model-method-moving average *k* for 74 participants of COVID-19 negative. In the previous experiment, we used the data of COVID-19 confirmed cases for only 3 weeks. In contrast, in this experiment, each method was applied to all the data submitted by 74 participants of COVID-19 negative. The reason is to maximize the sensitivity to anomaly detection for each method. In Fig. 5, we have each model-method combination on *x*-axis and the average number of detected anomalies from 74 participants on *y*-axis. Since each method is applied to a COVID-19 negative individual, the *y* value indicates the number of false positives. 74 participants of COVID-19 negative did not report any COVID-19 symptoms or underlying disorders. Thus, the outliers from the data of 74 participants could be regarded as false positives.

As shown in Fig. 5, RHR-OC-SVM reported the lowest number of false-positive detection from the same 74 participants. Note that HROS-OC-SVM in the previous section shows the best performance in most comparisons, but it reports the most false positive in this experiment. In addition, RHR-MD reports similar false positives with HROS-OC-SVM, and HROS-MD reports relatively few false positives compared with RHR-MD and HROS-OC-SVM. This experiment could show that RHR-OC-SVM is not better than HROS-OC-SVM for detecting COVID-19 confirmed cases, but it makes fewer false positives. Thus, RHR-OC-SVM can offer better performance than all other techniques in reality. Similar to the results applied to the previous COVID-19 confirmed cases with varying the moving average size, we, from this experiment, could conclude t that RHR-OC-SVM suffers less from the over-fitting problem than the others.

### Overall comparison with another technique

**Table 4:** Comparison between 3 methods, MD-MCD, OC-SVM, isolation forest (Iso-Forest).

	Models/methods							
	RHR-MD	RHR-Iso-Forest$^+$	RHR-Iso-Forest$^*$	RHR-OC-SVM	HROS-MD	HROS-Iso-Forest$^+$	HROS-Iso-Forest$^*$	HROS-OC-SVM
Relative detection accuracy	66.20%	74.50%	73.20%	88.80%	73.40%	63.70%	69.51%	95.30%
The number of outliers (COVID-19 positive)	41.87	29.48	29.57	29.57	48.41	36.55	56.55	32.29
Early detection time	7 day 2 h (170 h)	8 day 23 h (215 h)	9 day 11 h (227 h)	11 day 19 h (283 h)	11 day (264 h)	12 day 9 h (297 h)	15 day 22 h (382 h)	11 day 12 h (276 h)
The minimum data collection time	3 day 7 h (79 h)	2 day 20 h (68 h)	2 day 20 h (68 h)	2 day 7 h (55 h)	2 day 8 h (56 h)	1 day 14 h (38 h)	1 day 14 hours (38 h)	1 day 14 h (38 h)
The number of outliers (COVID-19 negative)	49.82	43.42	51.78	39.96	43.41	50.85	65.26	53.26

** RHR-Iso-Forest$^+$ and HROS-Iso-Forest$^+$ set 0.1 to the contamination parameter and RHR-Iso-Forest$^*$ and HROS-Iso-Forest$^*$ set ’auto’ to the contamination parameter.

Table 4 extends the comparison between MD-MCD and OC-SVM methods, including Isolation Forest, from a broad point of view. The comparison is performed in three aspects: The relative detection accuracy, the number of outliers (COVID-19 Positive, COVID-19 Negative), the early detection time, and the minimum data collection time. The minimum data collection time refers to the minimum collection time of sufficient data for meaningful initial detection. The anomaly detection techniques in this paper do not require computation time nor GPU, an additional computation device, once they build a detection model after collecting sufficient training data. This means that anomaly detection can be performed immediately when sufficient training data is ready. The MD-MCD methods (RHR-MD and HROS-MD) are set to the same parameter values used in Mishara et al.^1^ and work as a baseline in this comparison. The OS-SVM method and the Isolation Forest method are set to the best parameter values showing the best performance. For example, the MD-MCD methods set the moving average to 400, but the others set it to 350. The Isolation Forest method sets the contamination parameters to 0.1 or ‘auto’. Those hyper-parameters for the best performance are selected by the try-and-error method and all evaluation results are presented through Supplementary Information. The Isolation Forest with ‘auto’ as the contamination parameter determines the contamination value as if the original paper on Isolation Forest^8,9^. The OC-SVM method sets 0.1 to $\nu$ and 0.15 to $\gamma$.

In this comparison, the number of outliers for the COVID-positive and negative cannot be used to evaluate the overall performance of the methods since none of them always outperform the others. However, the comparison in the aspects of the relative detection accuracy, the early detection time, and the minimum data collection time evidently shows that OC-SVM method outperforms the others, MD-MCD and Isolation Forest methods, in most cases. The relative detection accuracy including the Isolation Forest method is also computed based on the confusion matrix like Fig. 3a in our paper. The confusion matrix to compute the relative detection accuracy of methods including the Isolation Forest is also found from our Supplementary Information.

## Discussion

Our experiments showed that the anomaly detection using OC-SVM to detect COVID-19 infection provides better performance in three aspects: earlier and more detection, and fewer false positives. We showed that RHR-OC-SVM-350 and RHR-OC-SVM-300 detected outliers 96 h and 12 h, respectively, earlier than RHR-MD-400 and HROS-MD-400 using MD-MCD, and the detection rate for the COVID-19 confirmed is improved, 40% and 23.5%, respectively. We also observed that RHR-OC-SVM-350 and RHR-OC-SVM-300 better detect COVID-19 with fewer data. As shown in Fig. 3b, the relative anomaly detection accuracy of each technique for each other methods shows that those of RHR-MD-400 and HROS-MD-400 are 66.5% and 72.4%, respectively, but those of RHR-OC-SVM-300 and RHR-OC-SVM-350 are 85.6%, a better relative detection accuracy, w.r.t to other techniques. It implies that RHR-OC-SVM-300 and RHR-OC-SVM-350 can detect more COVID-19 infection cases. Figure 2b shows that RHR-MD-400 and HROS-MD-400 report detection of outliers 170 and 264 hours before reporting COVID-19 symptoms onset, whereas RHR-OC-SVM-350 and RHR-OC-SVM-300 method detects anomalies 283 hours before reporting COVID-19 symptoms onset, showing 113 hours and 19 hours faster detection performance than MD-MCD used by Mishara et al.^1^. In the ability to suppress false positives, as can be seen from Fig. 5, RHR-MD-400 reported 49.82 and HROS-MD-400 43.41 outliers for 74 COVID-19 negative participants, but RHR-OC-SVM-350 and RHR-OC-SVM-300 report relatively fewer false positives, with 39.96 and 40.36, respectively.

Conclusively, RHR-OC-SVM-350 and RHR-OC-SVM-300 reported more (23.5–40%) COVID-19 positive earlier (12 h-4 days) with fewer data and less false detection than others.

The initial learning period of the technique using HROS is shorter than that of the method using RHR since RHR data discards the data of the step more significant than 0. However, once learning using RHR data is over, it is observed that the technique using RHR is more accurate and agile in reporting COVID-19 outliers.

In using OC-SVM, the value of $\nu$ denotes the maximum ratio of data outside the hyper-plan as if MD-MCD is sensitive to the threshold value. We show how to determine $\nu$ and $\gamma$ values in the section of OC-SVM-based Detection. Both OC-SVM and MD-MCD produce an anomaly detection mode-specific solely for a single person. Thus, it is necessary to develop a novel model general enough for all cases.

Zhu et al.^10^ compares the detection accuracy of smartphone-reported symptoms and self-reported symptoms, showing that the COVID-19 detection by smartphone-reported symptoms is more accurate (0.09 in AUC) than self-reported symptoms in detecting COVID-19. However, to the best of our knowledge, no literature discusses the fatality of COVID-19 detected by smartphone-reported symptoms.

## Related work

Medical diagnosis using physiological information is one of the oldest and most popular diagnostic methods. For example, the time-series characteristics of heart rate are closely related to the prevalence of several diseases such as metabolic syndrome, diabetes, hypertension, and myocardial infarction^11^. Li et al.^12^ presented a Lyme disease prediction system that measures physiological indicators, such as abnormal blood oxygen concentration, heart rate, and skin, based on physiological data sampled over 250,000 a day through smartwatches from 43 people. It showed that Lyme disease and inflammation could be predicted through statistical analysis of temperature changes.

Recently, many researchers have studied the use of physiological information obtained from wearable devices, such as a smartwatch, to predict diseases or infectious diseases and accurately understand patients’ current status in real-time. In particular, as sensors of wearable devices become cheaper than before, provide more accurate physiological information, and obtain participant data in real-time through various communication networks, wearable devices are being actively used for various diseases and health management. For example, Powers et al.^13^ introduced a monitoring system that was constructed by measuring 225 people for more than 6 months from wearable inertial sensor information to determine the dosage per patient with Parkinson’s disease. Kim et al.^14^ constructed a cardiovascular disease prediction model using wearable physiological information from 6170 people. They applied three models (logistic regression, artificial neural network, and support vector machine) to compare the suitability of the algorithm. As a result, the support vector machine-classified cardiovascular diseases and obtained well-predicted results from the test datasets.

In many cases, a mobile device, e.g. smartwatch, is used as a real-time physiological monitoring system to monitor health and disease status of the patients, such as Li et al.^15^, Kruizinga et al.^16^, Ding et al.^17^, Rodrigues et al.^18^, Gruwez et al.^19^, Perino et al.^20^, Avram et al.^21^, and Nasarre et al.^22^. Li et al.^15^ shows the validity of measurements by smartwatches for 6 Minutes Walking Tests and consistency between Walking distances measured by doctors and by smartwatches. Ding et al.^17^, and Perino et al.^20^ present the use of a smartwatch and application that demonstrate reasonable accuracy for Atrial fibrillation detection and high usability. Avram et al.^21^ describes the estimation of the sensitivity and specificity of atrial fibrillation in the user’s sinus rhythm in the user’s daily life using a smartwatch. Rodrigues et al.^18^ shows the usability of detecting the position of Alzheimer’s patients lost away. Gruwez et al.^19^ shows the usability of the AI algorithm analyzing continuous out-of-hospital PPG tracings from smartwatch annotating heartbeats by comparing it with continuous ECG monitoring, both during AF and non-AF rhythms in a heterogeneous patient population. Nasarre et al.^22^ explains how to prevent a heart attack and to detect sudden abnormal symptoms of the user by using the smartwatch’s electrocardiogram. Kruizinga et al.^16^ shows that the physiological data from smartwatches can be used as digital biomarkers, physical activity, and HR and shows promise for quantifying postdischarge recovery in a non-invasive manner, which can be helpful in pediatric clinical trials and clinical care.

In particular, one of the practical applications of smartwatches for disease diagnosis is to predict infectious disease prediction, such as COVID-19, which is of much value in such a global pandemic. Mishra et al.^1^ showed the applicability of physiological information from smartwatches for the pre-symptomatic detection of COVID-19. They used a classical time-series data analysis, Mahalanobis Distance computation, and dictated COVID-19 positive of participants with 63% of accuracy, based on heart rate and steps from smartwatches. Natarajan et al.^2^ obtained respiratory rate, heart rate, Root Mean Square of the Successive Differences (RMSSD), and entropy from 2745 out of 30,534 people who received PCR test by collecting respiratory rate, heart rate, and heart rate variability data from a COVID-19 proton smartwatch. The entropy data are used as training data to determine the severity of COVID-19 patients in 1D-CNN. As a result, the seriousness of 196 cases requiring hospitalization among 2745 points is accurately predicted in this paper. Similar to our approach, Bogu et al.^23^ have reported more advanced results. Instead of probabilistic, machine learning-based anomaly detection techniques, Bogu proposed a method to predict COVID-19 infection by detecting outliers in resting heart rate (when the step is 0) using Long Short Term Memory (LSTM) and Autoencoder. As a result, 23 out of a total of 29 people tested positive for COVID-19^23^.

Compared to Mishra et al.^1^, this paper shows the better performance of our approach using OC-SVM in three aspects: earlier and more detection, and fewer false positives. Compared to Bogu et al.^23^, this paper shows that OC-SVM can bound the minimum period of training OC-SVM to make accurate detection as shown in Fig. 4b.

## Methods

### Study design

All datasets we used in this paper are from the accessible datasets in https://www.nature.com/articles/s41551-020-00640-6. The datasets used in this paper are open access data approved by Standford University Institutional Review Board. The procedures used in this study followed the principles of the Declaration of Helsinki.

### Details of methods

In this paper, we present an optimization of the anomaly detection of pre-symptomatic COVID-19 detection through smartwatch by using machine learning (ML), i.e., One Class-Support Vector Machine (OC-SVM). Our approach to optimizing the anomaly detection using OC-SVM is carried out with comparing OC-SVM with Mahalanobis Distance-Minimized Covaraicen Determinant (MD-MCD) of Mishara et al.^1^ [In this paper, Mahalanobis Distance-Minimized Covaraicen Determinant (MD-MCD) is represented by MD in short]. Both methods create individual anomaly detection models solely for each participant.

In other words, the anomaly detection model built by the methods must be applied only for the participant who provided the data and cannot be applied for others.

Our optimization is performed in the following steps: First, OC-SVM is applied for the same data sets of Mishara et al.^1^. As a result, we show that OC-SVM has a better anomaly detection performance in that, from the same datasets of COVID-19, it has detected at sooner time points and found more the anomaly than the previous work^1^. We compare the two anomaly detection approaches: Mahalanobis Distance from Mishara et al.^1^ and OS-SVM that we propose. In particular, we use two different physiological models: Resting Heart Rate (RHR) and Heart Rate of Steps (HROS). Consequently, we compare four different combinations of models and methods: Resting Heart Rate (RHR)-Mahalanobis Distance (MD), One Class-Support Vector Machine (RHR-OC-SVM), Heart Rate of Steps (HROS)-MD, and HROS-OC-SVM. Those comparisons are in terms of (1) the number of outliers that could indicate a COVID-19 infection and (2) the distance between the occurrence date of the first outlier and the confirmation date of COVID-19 infection.

Second, we vary the length of data sampling periods, i.e., the moving average size of time-series data, in order to find the minimum sample size that is still as capable of detecting COVID-19 infection as the sample data size of the previous work^1^. Our goal is to find a smaller size of sample data that can detect COVID-19 and shorten the initial detection period of COVID-19.

Third, we compare the suppression of false positives of each approach. The exact anomaly detection should carry out not only early and exact detection with a small size of data but also the suppression of false positives. To show the suppression of false positives, we test each combination of models and methods for 74 non-COVID-19-infected ones and check how many outliers each technique produces for the same datasets of non-infected ones. The datasets used by our experiment are the COVID-19-related data from Mishara et al.^1^. The datasets that Mishara et al.^1^ collect are from 4652 participants who own a smartwatch. We utilize the datasets of 32 COVID-19 positive confirmed participants and 74 COVID-19 negative participants. The data of three participants from COVID-19 confirmed 32 participants are excluded from our experiment since one of the three participants has too small amount of data collected (less than 2 days) and the other two participants had not reported the onset of their COVID-19 symptoms until they were confirmed to be COVID-19 positive. The data are time series data consisting of the number of steps and heart rate on time, the time point of reporting COVID-19 symptoms onset, and the time point of COVID-19 confirmation. The time-series dataset for each participant is sampled every 4 s, 5 s, or 1 min. In our experiment, we use only heart rate and step data from COVID-19-positive patients. This experiment is performed using Scikit-learn.

The anomaly detection we adopt to this paper is performed as follows:

- Mishara et al.^1^ uses heart rate/steps as time series data. In particular, let the resting heart rate (RHR) be the resting heart rate when the number of steps is 0, and the heart rate of the resting heart rate and the heart rate when the number of steps is greater than 1 is referred to as Heart Rate Of Steps (HROS). As in Mishara et al.^1^, this paper also performed anomaly detection for COVID-19 detection using RHR and HROS.
- To calculate the time series mean of the data for one patient, the time series data are smoothed by a moving average (MA) and downsampling and the Z-score is further normalized.
- For patients, the moving average is calculated to calculate the time series average.
- We downsample the moving average of a patient to obtain the Z-score of the smoothed moving average.
- The propensity and residual values are taken from the Z-score and the additive method is used to correct the periodicity of the Z-score.
- Finally, the above Z-scores are normalized to mean 0 and variance to 1.
- With the above steps, anomaly detection is performed on time series data of individual COVID-19 patients.

### Mahalanobis distance-anomaly detection

Mahalanobis distance is a generalized distance measurement useful for determining the similarity between an unknown sample and a set of known samples in consideration of the correlation between variables. It is a multi-dimensional generalization of the idea of measuring how many standard deviations away a point *P* is from the mean of a distribution *D*. Mahalanobis distance *MD* is computed by

1 $$\begin{aligned} MD = \Big [(x_B- x_A)^T * C^{-1} * (x_B-x_A) \Big ]^{\frac{1}{2}}, \end{aligned}$$

where $x_A$ and $x_B$ is a pair of value points and *C* is the sample covariance matrix.

MD-MCD classifies out the outlier data that is over a threshold that is related to $\chi$-square. For the multivariate normal distribution with *p* random variables, the squared *MD* value is a $\chi$-square distribution with *p* degrees of freedom. When the square value of *MD* for the test data is calculated, it is defined as a threshold value using the quantile of the $\chi$-square distribution. In this study, the number of parameter is used $p = 2$ with the predetermined threshold is defined as the 90.0% quantile of a $\chi$-square distribution with 2 degrees of freedom. The Mahalanobis Distance problem is that when extreme outliers occur, it can mess up the covariance matrix and measure irrational distances The threshold is determined by $\chi$-square. The Mahalanobis Distance problem is that when extreme outliers occur, it can mess up the covariance matrix and measure irrational distances. For this reason, MD-MCD extracts and uses the data that can minimize the determinants of the covariance matrix of the data sample in finding the variance or mean of data. In this study, the threshold value is 0.1 and the data extraction ratio to minimize the determinants of the covariance matrix of the data sample is 0.7.

In our experiments, we use the class $\mathtt {sklearn.covariance.EllipticEnvelope}$ of scikit-learn packages^9^ to implement the anomaly detection method based on Mahalanobis Distance (MD) used in Mishara et al.^1^. As mentioned early, the threshold is set to 0.1, which is given by the argument $\mathtt {contamination}$ of the class, and the data extraction ration is set to 0.7, which is given by the argument $\mathtt {support\_fraction}$ of the same class.

### OC-SVM-based detection

Support Vector Machine (SVM) basically separates all the data points from the origin (in feature space F) and maximizes the distance from this hyper-plane to the origin. OC-SVM is a one-class classifier that introduces to SVM the maximum ratio of data outside the hyper-plane, denoted by $\nu$. The class $\mathtt {sklearn.svm}$ is a machine learning class providing SVM and OC-SVM as well. Constructing a hyper-plane needs to determine $\gamma$ that is a coefficient for Radial Basic Function (RBF) and Gaussian kernel. If $\gamma$ is large, the data close to the origin is considered as a support vector, otherwise, the data far from the origin is considered as a support vector. That is, the larger $\gamma$ is, the closer data to the origin compose a support vector. $\nu$ determines the maximum proportion of data outside the hyper-plane. In our setting, $\gamma$ is set to 0.15 and $\nu$ is set to 0.1.

**Figure 6:**
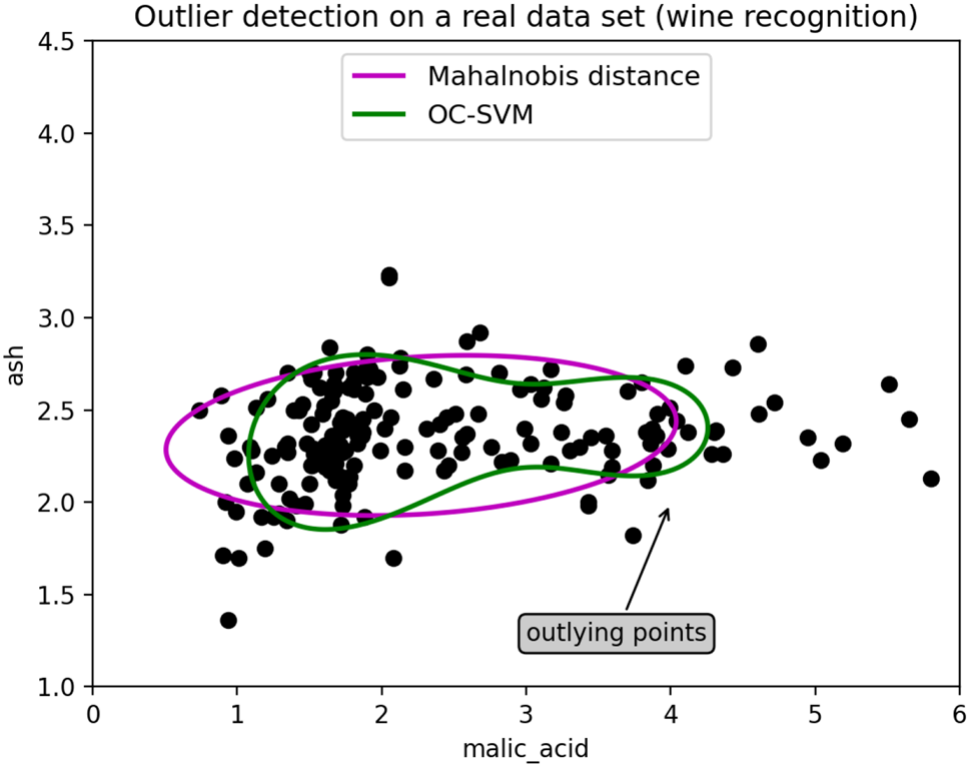
An example of anomaly detection using MD and OC-SVM (Wine recognition).

The parameters $\nu$ and $\gamma$ are set to specific values by the try-and-error method. We use three aspects of OC-SVM method to identify which values of $\nu$ and $\gamma$ show the best performances: The number of outliers, The early detection time, and the relative detection accuracy. The values of $\nu$ varies from 0.1 to 0.5 by increasing 0.1 and the value of $\gamma$ varies from 0.05 to 0.5 by increasing 0.05. As a result, 0.1 for v and 0.15 for Y show the best performance in the above three aspects. The testing results to identify the best values of parameters $\nu$ and $\gamma$ are found in our Supplementary Information. Figure 6 is an example data set for wine recognition provided by scikit-learn. The *x*-axis represents malic acid, and the *y*-axis represents ashes. The pink line is the boundary circle generated by the class $\mathtt {covariance.EllipticEnvelope}$ providing MD-MCD, and the green line is the boundary circle generated by the class $\mathtt {sklearn.svm}$ providing OC-SVM. The boundary circles created for each class serve as a criterion for distinguishing normal from abnormal. If the data points are inside the circle, they are labeled as normal. Otherwise, they are labeled as abnormal.

As shown in Fig. 6, the hyper-plane created by OC-SVM on the green line creates a more sophisticated boundary closer to the given data than the one created by Mahalanobis distance technique. It shows that OC-SVM distinguishes the boundaries of clustered points better than Mahalanobis Distance.

### Ethical approval and consent to participate

The original data used in this study were anonymised before its use and this study has no specific information on participants.

## Conclusions

The recent spread of the COVID-19 pandemic is mainly by asymptomatic patients. In particular, Quarantine in Korea, which focuses on controlling the cause of transmission through the transmission route, is inevitably helpless for these asymptomatic patients. The advanced medical functions and the cheapness make the smartwatch a valuable means for the preliminary medical diagnosis of such asymptomatic patients. Recently, the spreading of OMICRON, one strain of COVID-19, shows that early detection and diagnosis of COVID-19 is still vital in overcoming the pandemic situation of COVID-19.

This paper proposed an optimization of pre-symptomatic COVID-19 detection based on physiological data from a smartwatch using OC-SVM, a machine-learning-based anomaly detection. We showed that OC-SVM presents a better anomaly detection performance in three aspects than MD-MCD than the previous work of Mishara et al.^1^.

In particular, we conclude that RHR-OC-SVM-300 and RHR-OC-SVM-350 using OC-SVM are the most effective model-technique-moving average size combination than the other techniques.

## Supplementary Information

Supplementary Information.

## Data Availability

The original data we have used for this study are originally all available in https://www.nature.com/articles/s41551-020-00640-6, and the public access to the database is open. According to^1^, the de-identified raw heart rate, steps and sleep data used in this study can be downloaded from the study data repository (https://storage.googleapis.com/gbsc-gcp-project-ipop_public/COVID-19/COVID-19-Wearables.zip). Also, the data supporting our finding of the current study are online available from the corresponding author upon a reasonable request.
